# A decade of thyroidology

**DOI:** 10.1007/s42000-018-0068-7

**Published:** 2018-10-10

**Authors:** Petros Perros

**Affiliations:** 1 Newcastle upon Tyne Hospitals NHS Foundation Trust, Newcastle University, Newcastle upon Tyne, UK; 2 Department of Endocrinology, Level 6, Leazes Wing, Royal Victoria Infirmary, Newcastle upon Tyne, NE1 4LP UK

**Keywords:** Thyroid, Advances, Decade

## Abstract

Significant scientific progress has been achieved in the past decade in thyroidology driven by scholarly enquiry, unmet patient needs, and investment by the pharmaceutical and diagnostics industry. In this review, nine publications have been selected for their impact in pushing the frontiers of knowledge and understanding. They include new perspectives in the diagnosis, pathophysiology, epidemiology and management of thyroid cancer, understanding of thyroid hormone physiology, and new treatments for Graves’ orbitopathy.

## Introduction

Reflecting on advances made in thyroidology over the past decade is interesting and relatively easy. However, passing verdict on the most significant publications is a major challenge fraught with the risk of doing injustice to important work while overstating trivia. One approach is to base judgement on citations [1]. This is a well-established scientific method and lets one off the hook by transferring responsibility to an anonymous process with its own peculiarities and intricacies. A serious drawback is the time bias associated with citations, with older publications being more likely to be cited than recent ones. Another approach is to pick out highly respected journals with the most rigorous peer reviewers; what can possibly go wrong? Well, a number of such papers immediately come to mind from the not-so-distant past which simply did not stand up to the test of independent reproducibility and are now only remembered for the wrong reasons. That left the author with one option: to use his own subjective judgement based on impact on clinical practice and understanding of thyroid pathophysiology. Nine papers were selected that had been published in the past decade and which broadly cover the main topics of thyroidology (Table 1). [2–10]

**Table 1:** Selected publications in thyroidology in the past decade (in chronological order)

1. Atzmon G, Barzilai N, Surks MI, Gabriely I. Genetic predisposition to elevated serum thyrotropin is associated with exceptional longevity. J Clin Endocrinol Metab. 2009;94:4768–-4775 (2) 2. Marcocci C, Kahaly GJ, Krassas GE, Bartalena L, Prummel, M, Stahl M, et al., 2011 Selenium and the course of mild Graves'’ orbitopathy. N Engl J Med 364: 1920–-1931 (3) 3. Chudova D, Wilde JI, Wang ET, Wang H, Rabbee N, Egidio CM, et al., 2010 Molecular classification of thyroid nodules using high-dimensionality genomic data. J Clin Endocrinol Metab 95:5296–-5304 (4). 4. Lawrence MS, Stojanov P, Polak P, Kryukov GV, Cibulskis K , Sivachenko A, et al. 2013 Mutational heterogeneity in cancer and the search for new cancer associated genes. Nature 499: 214–-218 (5). 5. Russ G, Royer B1, Bigorgne C, Rouxel A, Bienvenu-Perrard M, Leenhardt L, 2013 Prospective evaluation of thyroid imaging reporting and data system on 4550 nodules with and without elastography. Eur J Endocrinol 168: 649–-655 (6). 6. Ito Y, Miyauchi A, Kihara M, Higashiyama T, Kobayashi K, Miyaat A 2014 Patients age is significantly related to the progression of papillary microcarcinoma of the thyroid under observation Thyroid 24: 27–-34 (7). 7. Werneck de Castro JP, Fonseca TL, Ueta CB, McAninch EA, Abdalla S, Wittmann G, et al., 2015 Differences in hypothalamic type 2 deiodinase ubiquitination explain localizedlocalised sensitivity to thyroxine. J Clin Invest 125: 769–-781 (8). 8. Ahn HS, Kim HJ, Kim KH, Lee YS, Han SJ, Kim Y, et al. Thyroid cancer screening in South Korea increases detection of papillary cancers with no impact on other subtypes or thyroid cancer mortality. Thyroid. 2016;26:1535–-1540 (9). 9. Smith TJ, Kahaly GJ, Ezra DG, Fleming JC, Dailey RA, Tang, RA, et al., 2017 Teprotumumab for thyroid-associated ophthalmopathy. N Engl J Med 376: 1748–-1761 (10).	

## Thyroid cancer and nodules

### The epidemic

In the past decade, we have witnessed a constant rise, and currently an exponential increase, in the incidence of thyroid cancer, which is affecting several countries, yet mortality has remained stable [11]. Increased detection has been proposed as the principal causative factor [12], although the latter explanation has been disputed [13, 14]. The most robust data that have explored this controversial issue come from South Korea, the country where the epidemic shows the most spectacular rise in incidence. Ahn and colleagues [9] performed an observational retrospective study, using national data, on the incidence of thyroid cancer, screening rates, and mortality in their country. It was observed that opportunistic screening for thyroid cancer by ultrasound crept into almost all routine practice in South Korea following the introduction of screening for breast and ovarian cancer, though screening rates for thyroid cancer varied in different regions. The authors were able to show a positive linear relationship between screening rates and diagnosis of thyroid cancer, while mortality remained low and unchanged. This work provides convincing evidence that screening of the general population for thyroid cancer leads to detection of small papillary thyroid cancers, which are largely of no clinical relevance. It lends support to the view that clinicians should exercise restraint before performing thyroid ultrasonography, which should only be undertaken when there is a clinical indication [15]. One question that remains unanswered is whether screening for thyroid cancer may be appropriate in selected subgroups. A significant proportion of patients who die from thyroid cancer are older people (often men) who present late with aggressive dedifferentiated cancers. Most of these are presumed to have a lengthy prodrome of occult differentiated thyroid cancer, which potentially presents a window of opportunity for detection and effective treatment.

### Genetic landscape

The Cancer Genome Atlas project was established in 2006 in the USA and has been generously funded with the aim of performing large-scale cancer genome sequencing. Lawrence et al. [5] studied 3087 tumour/normal tissue pairs across 27 tumour types, including papillary thyroid cancer. Whole-exome and whole-genome sequencing was used to study the samples. One of the novel aspects of the study was the development of an analytical methodology for dealing with false-positive results. The principal finding of interest from the point of view of thyroid cancer was that the rate of non-silent mutations was very low (0.41/Mb). It was thus determined that papillary thyroid cancers are genetically among the simplest of human cancers. The significance of this finding is that understanding the relationship between driver mutations, genes, histology and biological behaviour is within reach with relative ease. Such knowledge has the potential to improve diagnostic methods and personalise treatment for thyroid cancer.

### Papillary thyroid microcarcinomas

Papillary thyroid microcarcinomas are common [16, 17], associated with a good prognosis, [18, 19] and often treated with thyroid lobectomy. Investigators from the Kuma Hospital in Japan have approached the management of such patients in a more conservative manner (70). This study included patients with proven papillary microcarcinomas based on cytology who had no local or distant metastases, no aggressive cytology and no evidence of local invasion. Patients were offered immediate lobectomy or active surveillance (thyroid ultrasound at 6 months and then annually). More than 1000 patients have been followed up for about 10 years. The majority (nearly 90%) have remained stable, about 8% have shown increase in the size of the tumour and 3.8% developed cervical lymph node metastases. Patients who progressed were treated surgically. To date, no patient has died or developed distant metastases. Interestingly, older patients were less likely to progress than younger ones. It was estimated that active surveillance cost four times less than immediate lobectomy, while the study also documents the natural history of papillary microcarcinomas and confirms an indolent course for the majority. This knowledge is already impacting clinical practice and is reflected in recent guidelines [15]. There would be undoubted benefits in being able from the outset to predict which patients will progress, and further research in this area is under way.

### Thyroid ultrasound in risk stratification of thyroid nodules

The golden standard in the workup of thyroid nodules is fine needle aspiration cytology (FNAC); however, a significant proportion (about 25%, [20]) of FNACs are indeterminate and diagnostic surgery is often required. Ultrasound of the thyroid can provide information about the likelihood of malignancy of thyroid nodules. The TIRADS scoring system was developed to provide an objective risk stratification tool [21, 22]. On the other hand, while reliable in a research setting, it was not easily transferrable to routine clinical practice. Investigators from Paris modified TIRADS to a simpler version, which appeared to perform well in a retrospective series [23]. It was followed by a large prospective, single-centre study of more than 4000 nodules with corresponding cytology or histology [6]. Benign nodules were assigned a score of 2 and malignant a score of 5. The data showed a very high positive predictive value for a score of 5 and a very low risk of cancer in nodules with a score of 2. Furthermore, interobserver reproducibility was high and it was estimated that use of this diagnostic tool reduced the need for FNAC by a third. The importance of this study is that it provided a simple scoring system that performs well, can be learned relatively easily and can be applied effectively in clinical practice. Including this step in the diagnostic workup of thyroid nodules removes the need to proceed to FNAC in a significant proportion of patients. Its weakness is that those patients with a score of 3 or 4 continue to present a management challenge.

### Molecular markers in FNAC material

In another study, investigators from 10 academic centres in the USA utilised molecular markers on FNAC material for the diagnosis of thyroid cancer [4]. Messenger RNA was extracted and many thousands of transcripts were generated and analysed. Surgical specimens were used initially, followed by FNAC material, and finally a small prospective (*n* = 48) set of FNAC samples from patients with nodules, including some with indeterminate cytology (*n* = 24). The authors concluded that the test had a high negative predictive value and specificity. The markers used in this study remain undisclosed and the test is now commercially available. Since then, several other kits have come on the market. These tests are promising but require further validation for routine clinical use and at present appear to be overpriced.

## Hypothyroidism

### Thyroid hormone replacement

Doctors in several developed countries are seeing ever greater numbers of patients with a diagnosis of hypothyroidism who complain that their hypothyroid symptoms are not completely resolved with levothyroxine (T4) therapy. This seems to affect about 10% of hypothyroid patients [24, 25], including patients with apparently well-controlled hypothyroidism (TSH within the normal reference range, [26]), leading to the hypothesis that T4 monotherapy may not be capable of completely restoring thyroid status [27]. Combination therapy with T4 and liothyronine (T3) is equally ineffective [28], perhaps because of the pharmacokinetics of T3 resulting in peaks and troughs in serum levels. Understanding of this clinical conundrum at the molecular level remains unclear. Antonio Bianco’s group has addressed this dilemma through a series of elegant experiments using the thyroidectomised rat as a model [8]. Animals were rendered hypothyroid and replaced with T4 alone, T4 and T3 boluses or a continuous supply of T4 and T3. Serum TSH was normalised in all three groups; however, normal serum FT3, tissue levels of T3 and expression of T3-regulated genes were achieved only in the group of animals treated with a continuous supply of T4 and T3. They also showed that the mechanism behind these differences involves ubiquination of D2, which differs in the hypothalamus (the regulator of TSH secretion) as compared to the rest of the brain. The significance of these findings is that they provide a plausible explanation for why T4 monotherapy may not relieve all hypothyroid symptoms in patients. Clearly, further studies in humans are required to explore this further.

### The thyroid axis in the very elderly

It has been known for a long time that thyroid function declines with old age, independently of antithyroid autoantibodies and comorbidities [29, 30]. It has also been noted that mortality among people older than 85 years is lower in those with a TSH > 4.8 mU/L [31]. Atzmon G et al. [2] performed a study of 232 Ashkenazi Jewish people with a median age of 97 years. They were compared with a group of offspring (median age 67 years), the offspring’s spouses (median age 66 years) and an unrelated Ashkenazi Jewish group of people with a median age of 75 years. The median serum TSH of offspring of the very old group was shifted to the right of the control group, while that of the very elderly group was even further to the right. Further analysis showed a moderate heritability effect and associations between serum TSH in the very old and certain TSH receptor SNPs. This study confirms that subclinical hypothyroidism in very old age is not disadvantageous and may be partially genetically determined. Clinicians are advised to tolerate mild TSH elevation in people over the age of 80 years.

## Graves’ orbitopathy

### The IGF-1 receptor and Graves’ orbitopathy

It has been known for some time that IGF-1 receptors are expressed in orbital tissues [32] and that TSH and IGF-I have a synergistic effect on growth and proliferation of thyrocytes [33]. In one study, Graves’ IgG was able to stimulate hyaluran production by orbital fibroblasts, and this effect seemed to be mediated via the IGF-1 receptor [34]. Teprotumumab is an IGF-1 receptor blocker developed as an anticancer drug but which failed phase I trials for efficacy. Based on the above evidence that the IGF-1 receptor may be involved in Graves’ orbitopathy, a study was performed [10]. The study was a prospective, multicentre, randomised, placebo-controlled trial which recruited patients with moderate to severe disease with a clinical activity score > 4 and disease duration < 9 months. Eight infusions of teprotumumab were administered at 3-week intervals. Eighty-seven patients were studied. Response was assessed at 24 months. The overall response rate was far superior with teprotumumab compared to placebo. The most remarkable observation was that teprotumumab was associated with an average 3 mm reduction in proptosis. This level of efficacy on proptosis has never been seen with medical treatments and is of great interest, as it may eliminate the need for surgical decompression in suitable patients.

### Selenium and Graves’ orbitopathy

It has been suggested that free radical generation may play a role in the pathogenesis of Graves’ orbitopathy [35, 36]. Since selenoproteins act as antioxidants [37], a role for selenium as an immune modulator [38] is possible. This led the European Group On Graves’ Orbitopathy (EUGOGO) to perform a study comparing selenium supplements with placebo [3]. The study was prospective, multicentre and randomised and recruited patients with mild, active Graves’ orbitopathy (clinical activity score > 3), with a disease duration < 7 months. Fifty-four patients were randomised to selenium selenite 100 mcg bd and 50 to placebo. Patients were treated for 6 months. The overall responses were significantly better in the selenium group compared to placebo, including a marked improvement in quality of life. Furthermore, fewer patients treated with selenium progressed to more severe disease. This study is remarkable for several reasons: it was completed with minimum funding amounting to no more than a few thousand Euros to cover the cost of the drug and placebo; it shows efficacy of a treatment which is tolerated extremely well; the treatment costs 15–20 Euros per patient; because mild Graves’ orbitopathy is so much more common than other variants of Graves’ orbitopathy, the impact of this treatment at a population level is probably greater than that of any other intervention for Graves’ orbitopathy. The precise mechanism of action of selenium supplements continues to be unclear. Whether selenium may have a role in prevention of Graves’ orbitopathy is unknown and is currently under investigation. [39]

## The future

Speculating about future advances in thyroidology is to some extent safe and can be based on extrapolations from current trends. With regard to therapeutics, publications are anticipated of numerous on-going studies of tyrosine kinase inhibitors in advanced thyroid cancer. Immunotherapies for autoimmune thyroid disease are also emerging [40], and we may see a return of interest in thyroid hormone agonists [41]. A slow release T3 preparation is expected to become available and clinical studies in hypothyroidism are sure to follow [42]. TSH receptor inhibitors, both monoclonal antibodies and small molecules, will also feature [43]. In basic research, we can expect to learn more about the application of regenerative medicine in thyroidology [44] and we will hear a lot more about the role of thyroid hormones in dementia [45]. The general themes that are likely to prevail include personalised medicine (especially as applied to the management of thyroid cancer), data mining and artificial intelligence, all of which are already beginning to be introduced into thyroidology [46, 47].

The author has three major aspirations for the next 10 years. One is that clinicians will work towards reversing the tide of unnecessary investigations and treatments for thyroid nodules and incidental thyroid cancers, which has an enormous negative impact on patient care and wastes large amounts of resources. The second is that the high expectations about tyrosine kinase inhibitors in advanced thyroid cancer will eventually translate to clinically meaningful outcomes and that these drugs may become affordable. And finally and most importantly, the global elimination of iodine deficiency.
